# Delphi: A Democratic and Cost-Effective Method of Consensus Generation in Transplantation

**DOI:** 10.3389/ti.2023.11589

**Published:** 2023-08-23

**Authors:** Marjan Afrouzian, Nicolas Kozakowski, Helen Liapis, Verena Broecker, Luan Truong, Carmen Avila-Casado, Heinz Regele, Surya Seshan, Josephine M. Ambruzs, Alton Brad Farris, David Buob, Praveen N. Chander, Lukman Cheraghvandi, Marian C. Clahsen-van Groningen, Stanley de Almeida Araujo, Dilek Ertoy Baydar, Mark Formby, Danica Galesic Ljubanovic, Loren Herrera Hernandez, Eva Honsova, Nasreen Mohamed, Yasemin Ozluk, Marion Rabant, Virginie Royal, Heather L. Stevenson, Maria Fernanda Toniolo, Diana Taheri

**Affiliations:** ^1^ Department of Pathology, John Sealy School of Medicine, University of Texas Medical Branch at Galveston, Galveston, TX, United States; ^2^ Department of Pathology, Medical University of Vienna, Vienna, Austria; ^3^ Nephrology Center, Ludwig Maximilian University of Munich, Munich, Germany; ^4^ Department of Pathology, Sahlgrenska University Hospital, Gothenburg, Sweden; ^5^ Department of Pathology and Genomic Medicine, The Houston Methodist Hospital, Houston, TX, United States; ^6^ Laboratory Medicine Program, Toronto General Hospital, University Health Network (UHN), Toronto, ON, Canada; ^7^ Department of Pathology and Laboratory Medicine, Weill Cornell Medicine, New York, NY, United States; ^8^ Arkana Laboratories, Little Rock, AR, United States; ^9^ Department of Pathology and Laboratory Medicine, Emory University, Atlanta, GA, United States; ^10^ Department of Pathology, Université de Sorbonne, Assistance Publique—Hôpitaux de Paris, Hôpital Tenon, Paris, France; ^11^ New York Medical College, Valhalla, NY, United States; ^12^ Department of Pathology and Immunology, Baylor College of Medicine, Houston, TX, United States; ^13^ Department of Pathology and Clinical Bioinformatics, Erasmus University Center Rotterdam, Rotterdam, Netherlands; ^14^ Institute of Experimental Medicine and Systems Biology, RWTH Aachen University, Aachen, Germany; ^15^ Departamento de Patologia Geral, Instituto de Ciências Biológicas, Universidade Federal de Minas Gerais, Belo Horizonte, Brazil; ^16^ Department of Pathology, Koç University School of Medicine, Istanbul, Türkiye; ^17^ Department of Anatomical Pathology, NSW Health Pathology, Callaghan, NSW, Australia; ^18^ School of Medicine and Public Health, College of Health, Medicine and Wellbeing, The University of Newcastle, Callaghan, NSW, Australia; ^19^ Department of Pathology, School of Medicine, University of Zagreb, Zagreb, Croatia; ^20^ Department of Laboratory Medicine and Pathology, Mayo Clinic, Rochester, MN, United States; ^21^ AeskuLab Pathology and Department of Pathology, Charles University, Prague, Czechia; ^22^ Department of Pathology and Laboratory Medicine, King Fahad Specialist Hospital-Dammam, Dammam, Saudi Arabia; ^23^ Department of Pathology, Istanbul Faculty of Medicine, Istanbul University, Istanbul, Türkiye; ^24^ Department of Pathology, Necker-Enfants Malades Hospital, Université de Paris Cité, Paris, France; ^25^ Department of Pathology, Maisonneuve-Rosemont Hospital, University of Montreal, Montreal, QC, Canada; ^26^ Kidney Pancreas Transplantation, Instituto de Nefrología-Nephrology, Buenos Aires, Argentina; ^27^ Department of Pathology, Isfahan Kidney Diseases Research Center, Isfahan University of Medical Sciences, Isfahan, Iran; ^28^ Urology Research Center, Sina Hospital, Tehran University of Medical Sciences, Tehran, Iran

**Keywords:** Delphi, Banff, thrombotic microangiopathy, kidney, transplantation

## Abstract

The Thrombotic Microangiopathy Banff Working Group (TMA-BWG) was formed in 2015 to survey current practices and develop minimum diagnostic criteria (MDC) for renal transplant TMA (Tx-TMA). To generate consensus among pathologists and nephrologists, the TMA BWG designed a 3-Phase study. Phase I of the study is presented here. Using the Delphi methodology, 23 panelists with >3 years of diagnostic experience with Tx-TMA pathology listed their MDC suggesting light, immunofluorescence, and electron microscopy lesions, clinical and laboratory information, and differential diagnoses. Nine rounds (R) of consensus resulted in MDC validated during two Rs using online evaluation of whole slide digital images of 37 biopsies (28 TMA, 9 non-TMA). Starting with 338 criteria the process resulted in 24 criteria and 8 differential diagnoses including 18 pathologic, 2 clinical, and 4 laboratory criteria. Results show that 3/4 of the panelists agreed on the diagnosis of 3/4 of cases. The process also allowed definition refinement for 4 light and 4 electron microscopy lesions. For the first time in Banff classification, the Delphi methodology was used to generate consensus. The study shows that Delphi is a democratic and cost-effective method allowing rapid consensus generation among numerous physicians dealing with large number of criteria in transplantation.

## Graphical Abstract

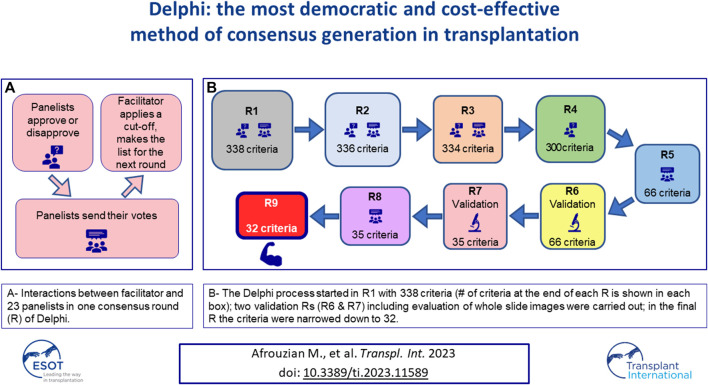

## Introduction

Transplantation is a relatively young and undoubtedly challenging science. In 1991, to address the main questions of organ transplantation a group of 20 experts composed of transplant clinicians/surgeons/pathologists gathered in Banff/Canada to build the Banff classification on allograft pathology [1]. Since then and for the past 30 years, experts have met every 2 years at Banff meetings, and generated many guidelines thankfully used by the Transplantation community. The Banff Working Group (BWG) for Thrombotic Microangiopathy (TMA) was formed in 2015 under the auspices of the Banff Foundation for Allograft Pathology to standardize criteria for diagnosing and classifying renal transplant TMA (Tx-TMA) [2]. In January 2016, a survey was circulated among the BWG participants regarding Tx-TMA. The results presented at the 2017 Banff conference, revealed considerable heterogeneity among nephropathologists regarding the criteria used for Tx-TMA diagnosis [3]. Therefore, standardization of diagnostic criteria deemed necessary. To achieve this goal, three phases were designed: Phase I (consensus among nephropathologists), Phase II (consensus among nephrologists), and Phase III (consensus of the consensus groups). The Delphi method of consensus generation was chosen to be used for the first time in Banff classification. Delphi is a structured process in which a panel of experts (the panelists) reaches consensus through iterative surveys with controlled feedback from the facilitator [4–7]. The panelists remain anonymous during surveys to ensure that their interactions remain devoid of biases that are usually introduced by group dynamics [4, 8, 9]. In addition, in contrast to other techniques like the nominal group technique or the NIH’s consensus conference, as the Delphi method does not require the physical presence of the participants in an actual meeting [10, 11], all interactions are designed to be online. The current work represents Phase I or the pathology phase of the study. Phase II, representing consensus among nephrologists, has already started and its results will be reported in the future. Phase I generated two interconnected papers that are being presented here. To omit redundancy, the results obtained from applying the Delphi method to transplantation, specifically to the diagnosis of Tx-TMA are reported in the current paper; in paper 2, published in the same issue [12], the pathology criteria themselves are being discussed in terms of their importance in the diagnosis of Tx-TMA in the practice of transplantation pathology.

## Materials and Methods

**FIGURE 1:**
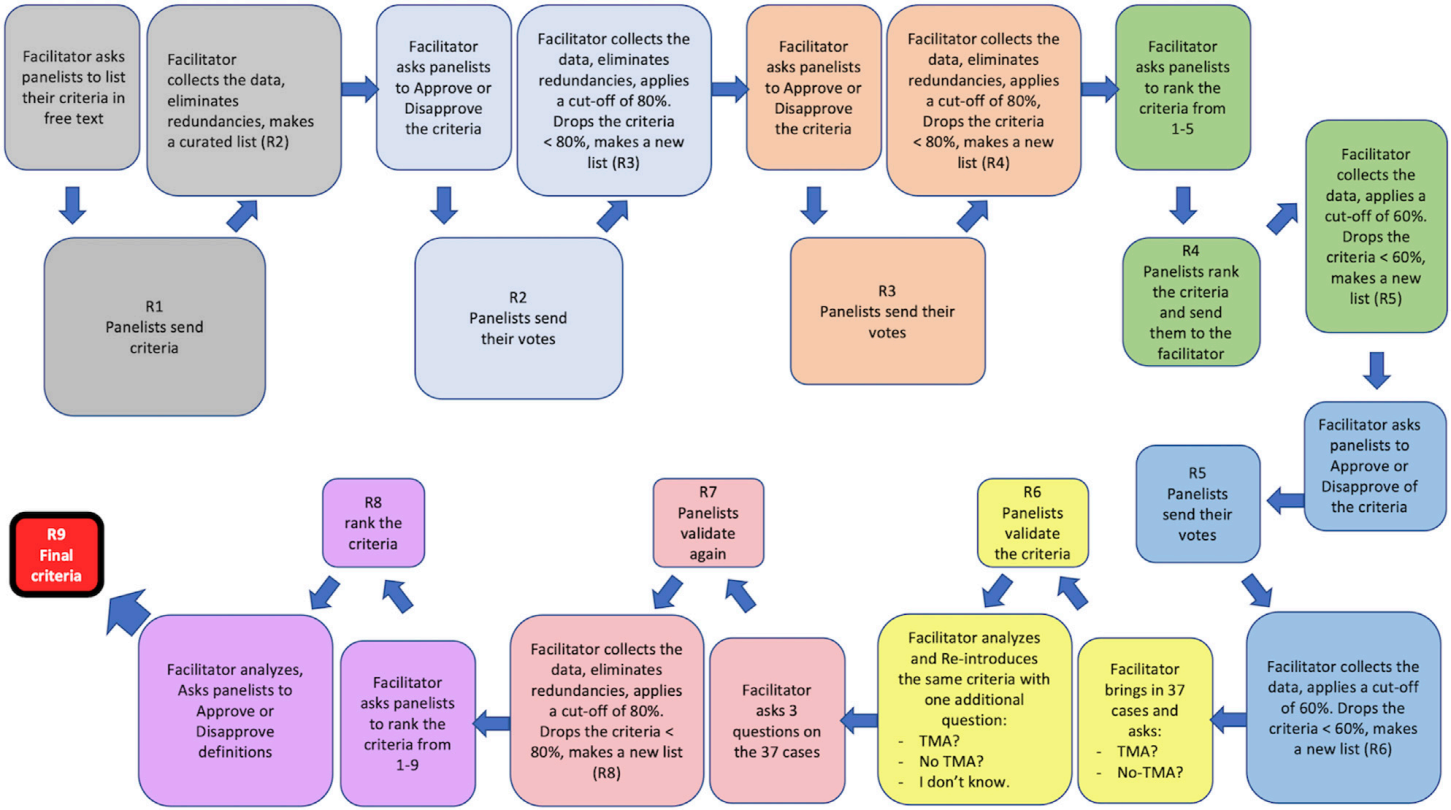
The Delphi process applied to this study. Nine rounds of survey (R1–R9) were designed. At the beginning of each round or R, the facilitator presented the panelists with the results (criteria) obtained from the previous R and asked them to either approve/disapprove of the listed criteria or to rank them. The panelists individually responded to this call and sent their votes to the facilitator who would collect the responses, eliminate redundancies, and apply a cut-off (80% or 60%) to that R. The results of the cut-off application were then shared with the panelists. A new list composed of all criteria that were above the cut-off was made by the facilitator and presented in the next R to the panelists. R6 and R7 were two rounds during which the criteria obtained from R5 were validated against 37 real-life cases by the panelists. R9 was a control round during which the integrity of the entire Delphi process was assessed. R9 was used to fine tune the definitions of the lesions that the panelists had difficulty with, during the validation R and was therefore called the Definition R.

Figure 1 illustrates the process of Delphi applied to this study. The pathological aspects of the material and methods are presented in paper 2 [12].

### Steering Committee and Panelists

A steering committee composed of two nephropathologists (MA, HL) performed literature review, identified areas of difficulty in Tx-TMA diagnosis and defined the terms “experts or panelists” by introducing inclusion and exclusion criteria, as required by Delphi [8]. Panelist was defined as a nephropathologist who had reported or published on Tx-TMA biopsies in the past 3 years (2012–2015). The steering committee members as well as the facilitator (MA) were excluded from the expert panel to avoid bias. Twenty-three nephropathologists from five continents met the above criterion and qualified as panelists.

### Design of the Delphi Rounds

To develop a core set of histopathological lesions (hereafter called “criteria”) a total of 10 rounds R) of survey (R0, R1 … R9) were launched at different points of the study, which spanned over a total of 5 years. Detailed information about each R and statistical analysis are provided below.

**R0:** The panelists were asked to send in free text their questions and areas of difficulty or ambiguity in the diagnosis of Tx-TMA. The panelists’ responses were shared with them at the end of R0. This survey was inserted based on the critique of the Delphi method by Keeney et al. and Diamond et al. [9, 10].

**R1:** The facilitator created a curated list of the criteria/opinions of the panelists and categorized them into positive (+) and negative (−) criteria. A positive criterion was defined as a criterion that, when present, would help the panelist make the diagnosis of TMA. A negative criterion was, by definition, a criterion which, when present, would help the panelist in ruling out the diagnosis of TMA. Based on the list obtained, 4 classes and 12 categories were formed: As shown in Supplementary Figure S1, the Pathology Class included six categories: Light microscopy positive (LM+); Light microscopy negative (LM−); Immunofluorescence microscopy positive (IF+); Immunofluorescence microscopy negative (IF-); Electron microscopy positive (EM+); and Electron microscopy negative (−). The Clinical Class comprised two categories: Clinical positive (Clin+); and Clinical negative (Clin−). The Laboratory Class included two categories: Laboratory positive (Lab+); and Laboratory negative (Lab−). Genetic criteria (Gen) were composed of tests that would help confirm the diagnosis of TMA. As some panelists had suggested a number of differential diagnoses, the facilitator also created a separate class for Differential Diagnosis Class (#D). Of note, TMA is a lesion with many mimickers. At the same time, different conditions may cause TMA. Therefore, the category of differential diagnosis included both mimickers and conditions that could cause TMA. After data collection and elimination of redundancies by the facilitator, the results were communicated to the panelists.

**R2:** Panelists were asked to either approve or disapprove of the results obtained from R1. Responses were collected, a cut-off of 80% called 80% agreement level (80%AL) was established by the facilitator: those criteria approved by 80% or more panelists were retained and the remaining criteria were held as potential candidates in the list that would be circulated in the next R. Results of R2 were shared with the panelists. In other words, an 80%AL would be, by definition, the level at which 80% of the participants would reach an agreement on a criterion. It is worth noting that according to the Delphi literature, the decision regarding the cut-off for each R, is totally arbitrary and can be changed from one R to another [6, 10, 13].

**R3:** Panelists were asked to approve or disapprove of the criteria including the differential diagnoses. A reasonable deadline was set, after which, the panelists’ R3 responses were collected. At this point, the facilitator eliminated redundancies, unified those criteria/opinions that were close in terms of meaning, and included in the same line terminologies that described the same phenomenon. These actions were taken to near opinions that were similar or at least not contradictory. The cut-off for this R was chosen to be 80% therefore, criteria approved by 80% or more panelists were retained and shared with the panelists. The remaining criteria approved by less than 80% of the panelists, were still shared with the panelists for the sake of transparency, however, were not included in the list circulated in the next R.

**R4:** A curated list of criteria was presented to the panelists who were asked to rank the criteria. The ranking was performed on a Likert-scale from 1 to 5 with anchors on 1 (highly suggestive of TMA), 2 (moderately suggestive of TMA), 3 (mildly suggestive of TMA), 4 (rather less favorable for diagnosis of TMA) and 5 (non-specific for diagnosis of TMA). After receiving all panelists’ responses, the mean rank for each criterion was calculated at one-decimal numbers. To make sure that no important criteria are dropped for the next R, the cut-off for this R was set at 60%. Criteria with mean ranks between 1 and 2.9 were considered being above the 60% cut-off and therefore were retained for the next R, while those with mean ranks between 3 and 5 were considered below the 60% cut-off and eliminated. Any criterion below the cut-off was also presented to the panelists at the end of R4 but dropped from the next R’s list. Based on the application by Jones et al [14], the facilitator provided feedback to panelists regarding all positive and negative criteria and the differential diagnoses.

**R5:** A curated list of criteria was presented to the panelists. To further narrow down the criteria, the panelists were asked to repeat the ranking of the criteria obtained from R4, using the scale of 1–5, with 1 being the most diagnostic criteria and 5 being the least favorable criteria. Responses were collected by the facilitator. To make sure that no important criteria are dropped for the next R, the cut-off for this R was set at 60% (as in R4); Mean ranks were calculated, and results shared with the panelists.

**R6:** This R was the first validation R. At this point, 37 cases collected and scanned by the facilitator were shared with the panelists who were asked to label the cases as either “TMA” or “No TMA.” Additionally, the panelists were asked to indicate which criterion on the list was used to make their diagnosis. For each biopsy the panelists ought to provide a mandatory comment about the case in free text, providing suggestions and criticizing the adequacy of the case or, the process. After receipt of all responses, facilitator and statistician analyzed R6 responses. Supplementary Table S1 reflects a snapshot of R6’s process. Comments not fitting in the “yes” or “no” responses were counted in a separate line called “N/A”. Based on the commentaries, it became clear that the R6 clearly needed to be re-designed, as some panelists were undecided regarding the diagnosis of some cases and could not decide if those cases were TMA or not. Therefore, the facilitator did not establish any cut-off for R6 and did not share the results of R6 with the panelists. To re-design the validation R, a third choice of “equivocal” (meaning I do not know) was added by the facilitator to the other two choices of “TMA” and “No TMA” and a new validation R called R7 was launched.

**R7:** In R7 panelists validated the criteria against the same 37 cases. During this R the panelists were asked to label each case as either “TMA,” “No TMA,” or “Equivocal.” Like R6, the panelists were asked to indicate which criteria on the list were used to make the final diagnosis on each case and enter their opinion in free text. After receipt of all responses, the facilitator and the statistician analyzed the responses. The cut-off for R7 was set at 80% i.e., a new analysis calculated the 80%AL for each of the 37 cases, and on each criterion. Criteria with <80% agreement were dropped for the next R. For clarification, the authors provide an example on criterion 1A here: in R7, the number of panelists who used criterion 1A for ANY of the 37 cases was counted. If out of 23 panelists, 19 or more (≥82.65%) used criterion 1A in at least 1 case, it was considered that criterion 1A was “used by more than 80% of the panelists” and therefore should be kept in the list for the next R. Results of R7 were shared with the panelists.

**R8:** The panelists were challenged in this R with the criteria obtained from R7 and asked to rank the criteria from 1 to n (1 being the most favorable criterion and n being the least favorable criterion), depending on the number of criteria in each category. Mean ranks of the criteria obtained from this R were calculated and shared with the panelists. This list contained the final criteria for diagnosis of Tx-TMA. It should be emphasized that R8 was originally planned to produce major and minor criteria by taking in to account panelists’ ranking. However, after examination of the results, the facilitator decided that future validation studies are needed to develop the concept of major/minor criteria.

**R9:** This R is usually used as a “control R” to assess the internal integrity of the process. The facilitator decided to use R9 to generate consensus on the definition of some lesions, that appeared to be morphologically problematic for some of the panelists during the previous Rs. Therefore, a consensus was needed regarding their definition. For example, the lesion “mesangiolysis”, an important diagnostic tool, did not receive sufficient vote in one of the rounds and was eliminated. The facilitator had to modify the cut-off for that round to keep this lesion as a criterion on the list. Therefore, R9 was called the definition R during which panelists were asked to define some terms used for a few light and electron microscopy criteria. All panelists had to provide in text format their own definition on these selected lesions. These definitions were then curated with elimination of redundancies, assembled in sentences by the steering committee, and shared with the panelists.

### Percentage Agreement (%A) and Percentage Agreement Levels (%AL)

Two terms were used to reflect the agreement between the panelists. The first term, %A, showed the agreement amongst the panelists concerning a diagnosis or criterion. The second term, %AL, reflected %A falling into a cut-off of agreement. For example, a 100%AL was the level on which 97%–100% of the panelists agreed on the same diagnosis on X number of cases. A 100%AL was therefore interpreted as ‘total agreement”. By the same token, a %AL was considered: poor if in the range of 0–40; fair if between 41 and 60; good if between 61 and 80; excellent if between 81 and 96 and total if between 97 and 100.

### Statistical Analysis

A detailed explanation of the statistical analysis is rendered below.

In R0 and R1 no statistical analysis was performed.

In R2 and R3, we calculated the approval percentage for each criterion based on the following formula:

$$\%approvalforcriterionk=\frac{\#ofparticipantsapprovedcriterionk}{\#ofparticipantsintheround}\times 100\%$$

In R4 and R5, we calculated the percentage of ranking based on the following formula:

$$\%rankingforcriterionk=\frac{averageranking\left(1to5\right)forcriterionk}{5}\times 100\%$$

In R6 and R7, we calculated the %A for each criterion based on the following formula:

$$\%agreementforcriterionk=\frac{\#ofparticipantsusedcriterionkinsomecases}{\#ofparticipantsintheround}\times 100\%$$

To assess the relative importance of the criteria, in R8, we calculated the percentage of favorable ranking based on the following formula:

$$\%offavorablerankingforcriterionk=\frac{\#ofparticipantsrankedcriterionk\left(1to6\right)}{\#ofparticipantsintheround}\times 100\%$$

All statistical modeling were performed using SAS, version 9.4 (SAS, Inc., Cary, NC). Some figures were drawn using the open source data visualization tool RAWGraphs [15].

## Results

Table 1 lists the original diagnoses of the 37 cases that were chosen to be validated (for panelists’ response, see below). The project started with 338 items/criteria obtained at the end of R1. Table 2 summarizes the evolution of the criteria from R1 to R9. By the end of R5, the facilitator was able to narrow down the items to 66 which included 56 criteria and 8 differential diagnoses. A list of the items entering R6 is provided in Supplementary Table S1. At the end of R7 the items were narrowed down to 35 including 27 criteria and 8 differential diagnoses. In R8, the number of items remained at 35. After R9, the facilitator eliminated three negative criteria that were expressed as “there is no criterion to help ruling out TMA.” These were eliminated because they could not be counted as criteria. Therefore, at the end of R9 the study ended up with 32 items including 24 criteria and 8 differential diagnoses. A detailed list of criteria and discussion about each criterion is outside the scope of this manuscript and will be published in the future.

**TABLE 1:** Diagnosis on the original 37 cases and percentage of agreement.

Cases	Original diagnoses	Panelists’ responses		% of agreement	
		TMA	No TMA	TMA	No TMA
1	TMA (diffuse)	19	4	83	17
2	TMA (focal) + ABMR	22	1	96	4
3	TMA (acute and chronic)	23	0	100	0
4	TMA (classical case)	22	1	96	4
5	TMA (classical case)	22	1	96	4
6	TMA (Early)	11	12	48	52
7	TMA found on EM only	8	15	35	65
8	TMA found on EM only	4	19	17	83
9	TMA (classical case)	22	1	96	4
10	ABMR + TMA	12	11	52	48
11	TMA (classical case)	19	4	83	17
12	No TMA (suspicious for ABMR)	7	16	30	70
13	No TMA (TCMR + C4d-neg ABMR)	5	18	22	78
14	Subtle TMA + CNI tox	14	9	61	39
15	TMA (classical case)	20	3	87	13
16	TMA (classical case)	17	6	74	26
17	TMA with rare thrombi	19	4	83	17
18	TMA with small thrombi	5	18	22	78
19	No TMA (GN with deposits)	4	19	17	83
20	TMA (acute and chronic)	22	1	96	4
21	TMA (acute and chronic)	21	2	91	9
22	TMA + Nephrosclerosis	18	5	78	22
23	No TMA (Chronic ABMR + TG + weak C4d+)	10	13	43	57
24	No TMA (Chronic ABMR + TG + weak C4d+)	6	17	26	74
25	TMA (classical case)	22	1	96	4
26	TMA (classical case)	21	2	91	9
27	TMA + Hypertensive arteriopathy	21	2	91	9
28	TMA (classical case)	23	0	100	0
29	TCMR	5	18	22	78
30	TMA (focal) + ABMR	12	11	52	48
31	TMA (classical case)	21	2	91	9
32	No TMA	12	11	52	48
33	TMA (classical case)	23	0	100	0
34	No TMA (recurrent MPGN)	14	19	42	58
35	No TMA (recurrent IgA glomerulopathy)	2	21	9	91
36	TMA (classical case)	23	0	100	0
37	TMA + ABMR	21	2	91	9

The original diagnoses of the 37 cases chosen to be validated for panelists’ response is shown along with the percentage agreement.

**TABLE 2:** Evolution of criteria from R1–R9.

Classes	R1	R2	R3	R4	R5	R6	R7	R8	R9
1. LM (LM+ & LM−)	90	89	87	85	16	16	12	12	11
2. IF (IF+ & IF−)	27	26	26	26	10	10	3	3	3
3. EM (EM+ & EM−)	43	43	43	32	5	5	5	5	4
4. Clin (Clin+ & Clin−)	55	55	55	52	12	12	3	3	2
5. Lab (Lab+ & Lab−)	70	70	70	70	9	9	4	4	4
6. Gen	16	16	16	14	4	4	N/A	N/A	N/A
Differential diagnosis	37	37	37	21	10	10	8	8	8
Total criteria	338	336	334	300	66	66	35	35	32

The number of criteria was narrowed down significantly during the Delphi process, starting from R1 and ending in R9. The table summarizes this evolution.

Supplementary Table S2 lists the number of the final criteria classified in each of the 12 categories which included 18 Pathological criteria (16 positive or 2 negative including 11 LM+, 1 IF+, 2 IF−, 4 EM + criteria); 2 Clinical criteria (2 Clin + criteria); 4 Laboratory criteria (including 4 Lab+ criteria). The 2 Lab- criteria were dropped because of insufficient votes (<20%). The process generated eight differential diagnoses entertained during the two validation Rs. Defining of eight criteria including 4 LM+ and 4 EM+ criteria emerged as a necessity at the end of R8. The panelists achieved this task during R9 which also served as a control R for the entire Delphi process.

### Agreement Among Panelists

**FIGURE 2:**
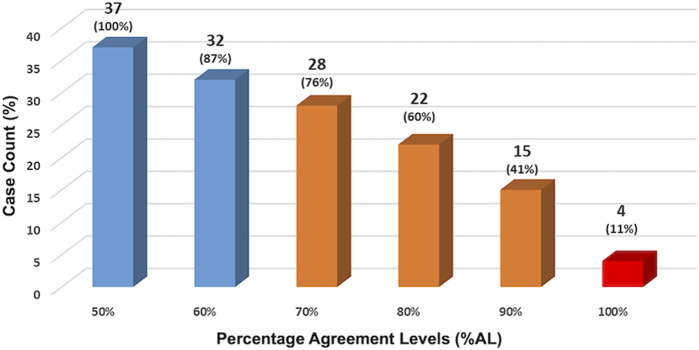
Panelists’ performance assessed at the end of R6. The facilitator observed the panelists’ performance looking at multiple agreement levels. At the end of R6, the first validation R, %AL was assessed at 50%, 60%, 70%, 80%, 90% and 100% levels. The results show that at 70%AL (middle bar), consensus was reached on 28/37 (76%) of cases. This means that almost three-quarters of the panelists agreed on three-quarters of the cases.

The facilitator observed the panelists’ performance looking at multiple agreement levels and at different points of the study. At the end of R6, the first validation R, %AL was assessed at 50%, 60%, 70%, 80%, 90%, and 100% levels (shown in Figure 2). The results show that at 70%AL (middle bar), consensus was reached on 28/37 (76%) of cases. This means that almost three-quarters of the panelists agreed on three-quarters of the cases.

A deeper look at the %AL at the end of the study is shown in Table 3 which shows the cumulative agreement levels among panelists and reveals that: 1- Total agreement (97–100%AL) was achieved in 4 cases (10.81% of cases; 2- Excellent agreement (81–100%AL) in 20/37 cases (54.05%); Good agreement (61–100%AL) in 31/37 cases (83.78%) and Fair agreement (41–100%AL) in all 37 cases (100%).

**TABLE 3:** Cumulative agreement levels among panelists.

Fair agreement 41–100%AL	Good agreement 61–100%AL	Excellent agreement 81–100%AL	Total agreement 97–100%AL
Obtained in 37/37 cases (100%)	Obtained in 31/37 cases (83.78%)	Obtained in 20/37 cases (54.05%)	Obtained in 4/37 cases (10.81%)
Case #:	Case #:	Case #:	Case #:
1, 2, 3, 4, 5, 6, 7, 8, 9, 10, 11, 12, 13, 14, 15, 16, 17, 18, 19, 20, 21, 22, 23, 24, 25, 26, 27, 28, 29, 30, 31, 32, 33, 34, 35, 36, 37	1, 2, 3, 4, 5, 7, 8, 9, 11, 12, 13, 15, 16, 17, 18, 19, 20, 21, 22, 24, 25, 26, 27, 28, 29, 31, 33, 34, 35, 36, 37	1, 2, 3, 4, 5, 8, 9, 11, 15, 17, 19, 20, 21, 25, 28, 31, 33, 35, 36, 37	3, 28, 33, 36

Different %ALs between the panelists regarding the diagnosis of the 37 validated cases: 41–100%AL, 61–100%AL, 81–100%AL and 97–100%AL.

## Discussion

### Delphi and Consensus

The term consensus is clarified in Delphi and defined as “general agreement, but not necessarily unanimity,” and includes the process to resolve objections by interested parties. A process would be considered a consensus, if all comments have been fairly considered, each objector has been advised of the disposition of his or her objection(s) and the reasons why, and the consensus body members have been given an opportunity to change their votes after reviewing the comments [16]. Delphi is a structured process of consensus generation in an iterative fashion through repeated anonymous surveys with controlled feedbacks given by the facilitator [6]. In Delphi a panel of experts (the panelists) can reach consensus through multiple online interactions, that would prevent introduction of bias from group dynamics. In contrast to other techniques like the nominal group technique or the NIH’s consensus conference, the Delphi method does not require the physical presence of the participants in an actual meeting [11].

### Comparing the NIH Type of Consensus Generation With Delphi

To compare the NIH type of consensus with the Delphi method, and why the Delphi method is preferred in some situations, a point-by-point description of both methods is presented below.

#### The NIH Type of Consensus Generation

The reader of the current paper is most probably familiar with the rules of the usual NIH type consensus generation. In this type of consensus: 1. Opinions/questions/criteria are usually pre-designed by a steering committee composed of the most experienced members of the group at the beginning of the process; 2. The literature has already covered some information about the incidence and/or definitions of the criteria/lesions and all panelists are on the same page; 3. Communications are in person or through online video-conferencing, therefore, the identity and opinions of the panelists, the most experienced, the less famous, the loudest and the silent, the most and least popular members of the group are known by all participants, introducing “human interaction bias” into the process. Therefore, “discussions” in this consensus model are performed by directly addressing one or multiple panelists and accepting or not an argument, *in situ* and within the group; 4. The criteria or the pathology cases brought to the consensus are the “subjects” of the study. This means that in the NIH model, the study is expected to validate the criteria with a significant number of cases, report *p*-values and inter-correlation coefficients (ICC), which evaluate criteria performance when put to test.

#### The Delphi Method of Consensus Generation

Delphi, however, has a fundamentally different approach to the panelists and the criteria. In Delphi: 1. Questions or criteria are not set in advance by the steering committee and the entire group of panelists set the tone by expressing their own opinions/questions/criteria at the beginning of Delphi; 2. Definitions of the criteria/lesions are not known at the start of the process as no one knows which lesions are going to reach the finish line. For example, this study started with 338 criteria and lesions suggested by the panelists. It is obvious that the steering committee could not possibly define all the 338 criteria at the beginning of the study, as this would introduce an external bias. Hence, such interventions from the steering committee or the facilitator are strictly prohibited during Delphi, allowing a democratic process devoid of any peer pressure, interference, and bullying. All 338 criteria had to enter R1, and those reaching the finish line by R8 were the result of a vigorous election process; 3. In Delphi, the “subjects” are the panelists, not the criteria nor the validated cases, therefore, *p*-values and ICCs are not expected to be generated; 4. All opinions are expressed anonymously, not only to eliminate peer pressure but also to allow a different type of “discussion.” To expand on this notion, it suffices to mention that in Delphi, the cognitive exercise starts with the first Rs when each panelist faces the list of criteria voted by other panelists, permitting self-reflection on personal knowledge, opinion, and experience. Later, in-mid process, after multiple Rs of voting and elimination of the criteria that have not received enough vote, a cognitive connection is automatically established between this panelist and the rest of the group creating a collective mind ready to validate the final list.

Results show that 3/4 of the panelists agreed on the diagnosis of 3/4 of cases. Comparing these results to the results of similar studies that used the NIH-type of consensus, one can draw the following conclusion regarding the quality of consensus: our results are comparable to other studies even though different methodologies and statistical analyses were used. For example, Liapis et al. reported that consensus was reached among about 75% of the pathologists who agreed on 75% of cases when scoring the number of glomeruli present in implantation (donor) biopsies. The consensus was below 75% when scoring was performed on glomerulosclerosis and other parameters such as number of arteries, and tubular atrophy (Data obtained from table 2, ICC results, Liapis H et al, AJT 2017) [17]. Although the quality of consensus appears to be similar in both studies, it is worth mentioning that the present study generated consensus on criteria and on diagnoses, not just on a single factor such as number of glomeruli. Therefore, a much complex consensus process was applied to our Delphi-based study.

### Performance of the Panelists

When analyzing panelists’ performance, the results are encouraging: a “good” level of agreement, was obtained on 31 cases, and consensus was reached among 70% of panelists on 28/37 (76%) of cases, basically implying that about three-quarters of the panelists agreed on three-quarters of the cases. This result shows that the Delphi process was able to generate an acceptable level of consensus among our panelists.

### Novelty

The use of Delphi as a consensus building method started in the last decade of the 20th century and, therefore, has been used by some disciplines for years. However, its introduction to the world of pathology is recent [18, 19], moreover, it has never been used in Banff classification. Furthermore, the novelty of our study is in the integration of a classical histopathology workup into the Delphi process, including interpretation of digital whole slide images accompanied by clinical history and laboratory data. This approach which is a modification of the usual Delphi method can be used in medicine, especially in transplantation pathology, where criteria generated during multiple consensus rounds could be validated against real-life cases. This modified Delphi method is, therefore, adapted to the needs of the pathology consensus process.

As in other methods, Delphi is partly an exercise to educate a group of participants to think and re-think about their definitions/cutoffs, adapting alternative terminologies in the process (in this case histopathologic criteria) and running the risk of less than 100% agreement. The latter, however, is not unexpected in an observational discipline, like histopathology, thus agreement cutoffs have to be introduced and are generally valid. Finally, the world has changed since the initiation of consensus building on allograft pathology and the creation of the Banff classification. Pandemic-related travel and contact restrictions, financial constraints, and global warming concerns—also related to academic air travel—all advocate for a revision of old practices. In this perspective, the Delphi methodology represents a great solution for consensus building in general and the Banff Classification operating through consensus in particular.

In conclusion, the Delphi methodology is a method of consensus generation that has not been used in Transplantation. For the first time in Banff classification, and in the Phase I of the study, Delphi was used by the TMA-BWG to generate consensus on MDC for TMA in renal allograft biopsies. We adapted the Delphi methodology to the needs of consensus building in pathology by using digital imaging during validation Rs. Delphi proved to be a highly efficient method of consensus generation among pathologists. The novelty of the study is in its anonymous yet democratic approach, online implementation, low cost, and ability to reach many participants from around the globe.

## Data Availability

The original contributions presented in the study are included in the article/Supplementary Material, further inquiries can be directed to the corresponding author.

## Supplementary Material

Supplementary Figure S1 Classification of the initial suggested criteria. The panellists were initially asked to suggest their criteria for diagnosis of Tx-TMA. After 338 opinions/ criteria were collected, the facilitator classified them into four classes including Pathologic, Laboratory and clinical criteria and differential diagnoses. Each of the classes were further divided into positive and negative categories, for example the pathologic criteria were divided into LM+, LM−, IF+, IF−, EM+ and EM− categories.

Supplementary Table S1 A snapshot of R6’s process. After receipt of all responses, facilitator and statistician analyzed R6 responses. This is a snapshot from the excel sheet that showed the result of the analysis on a few cases (labelled as KB1, KB2, …etc.). In the left column, the validation questions are shown including question 1 (Is this a case of TMA or No TMA?), and question 2 (Please mark the criteria—listed below—which helped you in your final diagnosis).

Supplementary Table S2 Number of final criteria obtained at the end of the study. The final criteria were classified in to 12 categories which included 18 Pathological criteria (16 positive or 2 negative including 11 LM+, 1 IF+, 2 IF−, 4 EM+ criteria); 2 Clinical criteria (2 Clin+ criteria); 4 Laboratory criteria (including 4 Lab+ criteria). The 2 Lab− criteria were dropped because of insufficient votes (<20%). The genetic category remained without any criteria due to lack of sufficient information. The process generated eight differential and the definition of eight criteria including 4 LM+ and 4 EM+ criteria was refined through consensus in R9.

## Abbreviations

ABMR, antibody-mediated rejection; (a)HUS, (atypical) hemolytic uremic syndrome; BWG, Banff Working Group; Clin, clinical data; CNI, calcineurin inhibitor; EM, electron microscopy; #D, differential diagnosis; DIC, disseminated intravascular coagulation; EC, endothelial cells; Gen, genetic criterion; GN, glomerulonephritis; HTN, hypertension; ICC, intraclass correlation; IF/IHC, immunofluorescence microscopy/immunohistochemistry; IgAN, IgA nephropathy; Lab, laboratory; LDH, lactate dehydrogenase; LM, light microscopy; MPGN, membranoproliferative GN; MDC, minimum diagnostic criteria; NT-ABMR, non-TMA-associated antibody-mediated rejection; PI GN, post-infectious glomerulonephritis; R, round; SLE, systemic lupus erythematosus; TTP, thrombotic thrombocytopenic purpura; TMA, thrombotic microangiopathy; Tx-TMA, transplant TMA; %A, percentage agreement; %AL, percentage agreement level.
