# Supplementary figures and images for: Delphi: A Democratic and Cost-Effective Method of Consensus Generation in Transplantation
Source: Transpl Int. 2023 Aug 23;36:11589. doi: 10.3389/ti.2023.11589 (PMC10481336; doi:10.3389/ti.2023.11589)

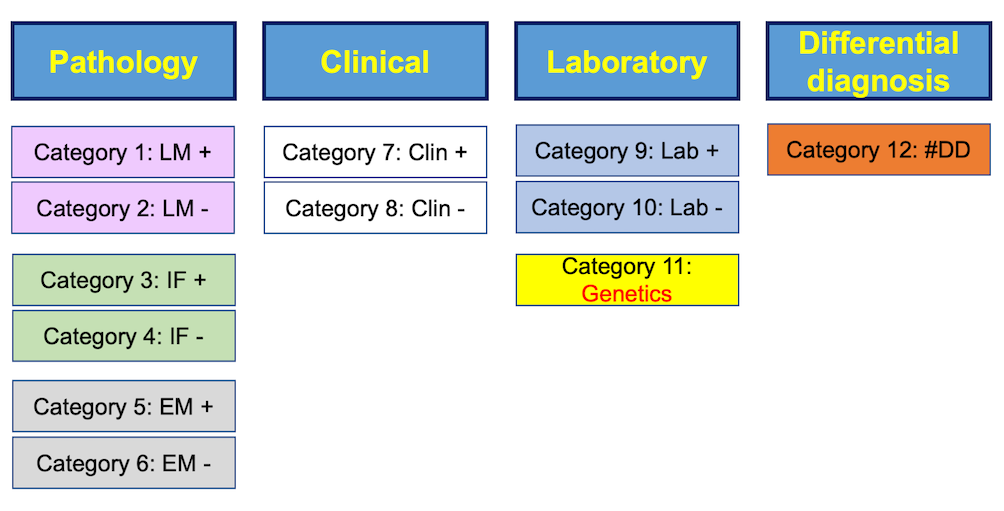

Supplement: Supplementary file 1 [file Image1.TIFF]
