# Supplementary material for: Delphi: A Democratic and Cost-Effective Method of Consensus Generation in Transplantation
Source: Transpl Int. 2023 Aug 23;36:11589. doi: 10.3389/ti.2023.11589 (PMC10481336; doi:10.3389/ti.2023.11589)
Supplement: Supplementary file 2 [file Table1.DOCX]

**Supplemental table S1.**

|  | **Summary** |  |  |  |  |  |  |  |  |  |  |
| --- | --- | --- | --- | --- | --- | --- | --- | --- | --- | --- | --- |
|  |  |  |  |  |  |  |  |  |  |  |  |
|  |  |  | **KB-10** | **KB-11** | **KB-13** | **KB-15** | … | **ST-1** | **ST-2** | **ST-3** | **ST-4** |
|  | Case was TMA |  |  |  |  |  | … |  |  |  |  |
|  | Case was not TMA |  |  |  |  |  | … |  |  |  |  |
|  |  |  |  |  |  |  | … |  |  |  |  |
| Question 1 | What is your diagnosis |  |  |  |  |  | … |  |  |  |  |
|  | TMA |  | 17,39% | 4,35% | 0,00% | 4,35% | … | 39,13% | 91,30% | 0,00% | 8,70% |
|  | Not TMA |  | 0,00% | 0,00% | 0,00% | 0,00% | … | 0,00% | 0,00% | 0,00% | 0,00% |
|  |  |  |  |  |  |  | … |  |  |  |  |
|  |  |  |  |  |  |  | … |  |  |  |  |
|  |  |  |  |  |  |  | … |  |  |  |  |
| Question 2 | Category I - LM + (LM lesion that its presence in the biopsy helped you make the diagnosis of TMA) |  | **KB-10** | **KB-11** | **KB-13** | **KB-15** | … | **ST-1** | **ST-2** | **ST-3** | **ST-4** |
|  | 1A - fibrin thrombi in glomerular hilum (HE, TCR, PAS, AFOG, MSB) |  | 65,22% | 95,65% | 0,00% | 95,65% | … | 43,48% | 8,70% | 100,00% | 91,30% |
|  | 1B - fibrin thrombi in glomerular capillaries (HE, TCR, PAS, AFOG, MSB) |  | 0,00% | 8,70% | 26,09% | 21,74% | … | 13,04% | 4,35% | 43,48% | 56,52% |
|  | 1C - thrombosed arterioles with fibrinoid change (HE, MSB) |  | 0,00% | 0,00% | 21,74% | 4,35% | … | 8,70% | 0,00% | 34,78% | 13,04% |
|  | 1D - fibrin thrombi in small arteries (TCR) |  | 4,35% | 21,74% | 4,35% | 17,39% | … | 4,35% | 4,35% | 17,39% | 21,74% |
|  | 1E - fibrin thrombi in glomerular subendothelial region (HE, TCR, PAS, AFOG, MSB) |  | 0,00% | 0,00% | 8,70% | 0,00% | … | 4,35% | 4,35% | 0,00% | 0,00% |
|  | 1F - thrombosed arterioles with recanalization (HE, MSB) |  | 0,00% | 0,00% | 0,00% | 0,00% | … | 0,00% | 0,00% | 0,00% | 0,00% |
|  | 1G - arteriolar platelet thrombi (CD61) |  | 0,00% | 13,04% | 73,91% | 4,35% | … | 4,35% | 0,00% | 4,35% | 0,00% |
|  | 1H - arterial intimal edema/mucoid changes (HE) |  | 0,00% | 4,35% | 4,35% | 8,70% | … | 0,00% | 0,00% | 8,70% | 4,35% |
|  | 1I - platelet thrombi in glomerular capillaries (CD61) |  | 13,04% | 21,74% | 69,57% | 8,70% | … | 17,39% | 8,70% | 26,09% | 17,39% |
|  | 1J - arteriolar subendothelial edema/intimal edema or mucoid changes |  | 0,00% | 4,35% | 91,30% | 8,70% | … | 4,35% | 0,00% | 0,00% | 0,00% |
|  | 1K - arteriolar onion skin change (HE, PAS, PAMS, TCR, VVG) (subacute change) |  | 26,09% | 8,70% | 39,13% | 4,35% | … | 52,17% | 8,70% | 17,39% | 21,74% |
|  | 1L - double contours (PAMS, PAS) (late lesion) |  | 0,00% | 0,00% | 73,91% | 0,00% | … | 8,70% | 0,00% | 4,35% | 0,00% |
|  | 1M - onion skin pattern in small arteries (HE, PAMS) |  | 21,74% | 4,35% | 4,35% | 4,35% | … | 30,43% | 86,96% | 4,35% | 8,70% |
|  | N/A |  | 0,00% | 0,00% | 0,00% | 0,00% | … | 0,00% | 0,00% | 0,00% | 0,00% |
|  |  |  |  |  |  |  | … |  |  |  |  |
|  | Category 2- LM - (LM lesion that its presence in the biopsy helped you rule out the diagnosis of TMA) |  | **KB-10** | **KB-11** | **KB-13** | **KB-15** | … | **ST-1** | **ST-2** | **ST-3** | **ST-4** |
|  | 2A - *there are no LM findings that can help ruling out TMA |  | 8,70% | 0,00% | 0,00% | 0,00% | … | 4,35% | 8,70% | 0,00% | 4,35% |
|  | 2B - Diffuse proliferative (exudative) glomerulonephritis pattern |  | 0,00% | 0,00% | 0,00% | 0,00% | … | 0,00% | 0,00% | 0,00% | 4,35% |
|  | 2C - spikes (PAMS) and epi-membranous fuchsinophilic deposits (TCR) |  | 52,17% | 65,22% | 60,87% | 56,52% | … | 52,17% | 43,48% | 65,22% | 56,52% |
|  | N/A |  | 0,00% | 0,00% | 0,00% | 0,00% | … | 0,00% | 0,00% | 0,00% | 0,00% |
|  |  |  |  |  |  |  | … |  |  |  |  |
|  | Category 3 - IF + (IF lesion that its presence in the biopsy helped you make the diagnosis of TMA) |  | **KB-10** | **KB-11** | **KB-13** | **KB-15** | … | **ST-1** | **ST-2** | **ST-3** | **ST-4** |
|  | 3A - glomerular intraluminal staining with fibrin-related antigens |  | 0,00% | 4,35% | 0,00% | 4,35% | … | 0,00% | 0,00% | 0,00% | 0,00% |
|  | 3B - glomerular sub-endothelial (mural) with fibrin-related antigens |  | 0,00% | 0,00% | 4,35% | 0,00% | … | 0,00% | 0,00% | 0,00% | 0,00% |
|  | 3C - linear or granular fibrin deposition within arteriolar/arterial lumen or wall |  | 0,00% | 0,00% | 0,00% | 0,00% | … | 0,00% | 0,00% | 0,00% | 0,00% |
|  | 3D - glomerular mesangial staining with fibrin-related antigens |  | 13,04% | 17,39% | 13,04% | 8,70% | … | 0,00% | 0,00% | 8,70% | 4,35% |
|  | 3E - absence of immune complex deposits |  | 13,04% | 13,04% | 13,04% | 8,70% | … | 0,00% | 0,00% | 8,70% | 4,35% |
|  | 3F - absence of immune complexes in glomeruli |  | 0,00% | 4,35% | 0,00% | 0,00% | … | 21,74% | 0,00% | 0,00% | 0,00% |
|  | 3G - IgM, C3 and C1q in areas of sub-endothelial expansion |  | 0,00% | 0,00% | 0,00% | 0,00% | … | 4,35% | 0,00% | 0,00% | 0,00% |
|  | 3H - segmental IgM and/or C3, although nonspecific (seen in TMA with segmental glomerulosclerosis) |  | 86,96% | 82,61% | 82,61% | 86,96% | … | 73,91% | 100,00% | 91,30% | 95,65% |
|  | N/A |  | 0,00% | 0,00% | 0,00% | 0,00% | … | 0,00% | 0,00% | 0,00% | 0,00% |
|  |  |  |  |  |  |  | … |  |  |  |  |
|  | Category 4 - IF - (IF lesion that its presence in the biopsy helped you rule out the diagnosis of TMA) |  | **KB-10** | **KB-11** | **KB-13** | **KB-15** | … | **ST-1** | **ST-2** | **ST-3** | **ST-4** |
|  | 4A - *there are no IF findings that could help ruling out TMA |  | 0,00% | 0,00% | 0,00% | 0,00% | … | 4,35% | 4,35% | 0,00% | 0,00% |
|  | 4B - suspected thrombi strongly positive for IgG/IgA/C1q/C3/IgM/Light chain |  | 0,00% | 0,00% | 0,00% | 0,00% | … | 0,00% | 56,52% | 0,00% | 4,35% |
|  | 4C - IgA predominance in the absence of TMA |  | 52,17% | 56,52% | 60,87% | 69,57% | … | 60,87% | 39,13% | 69,57% | 65,22% |
|  | N/A |  | 0,00% | 0,00% | 0,00% | 0,00% | … | 0,00% | 0,00% | 0,00% | 0,00% |
|  |  |  |  |  |  |  | … |  |  |  |  |
|  | Category 5- EM + (EM lesion that its presence in the biopsy helps you make the diagnosis of TMA) |  | **KB-10** | **KB-11** | **KB-13** | **KB-15** | … | **ST-1** | **ST-2** | **ST-3** | **ST-4** |
|  | 5A - Fibrin tactoids in the lumen or in the widened (expanded) sub-endothelial space (glomerular or vascular) |  | 60,87% | 39,13% | 95,65% | 56,52% | … | 0,00% | 0,00% | 0,00% | 0,00% |
|  | 5B - *sub-endothelial widening/rarefaction and accumulation of "fluff" |  | 13,04% | 13,04% | 30,43% | 26,09% | … | 0,00% | 0,00% | 0,00% | 0,00% |
|  | 5C - glomerular platelets aggregates |  | 8,70% | 13,04% | 26,09% | 8,70% | … | 0,00% | 0,00% | 0,00% | 0,00% |
|  | 5D - GBM duplication/ lamination/multilayering with mesangial (or mesangial cell) interposition (late lesion) |  | 39,13% | 43,48% | 4,35% | 13,04% | … | 100,00% | 100,00% | 100,00% | 100,00% |
|  | N/A |  | 0,00% | 0,00% | 0,00% | 0,00% | … | 0,00% | 0,00% | 0,00% | 0,00% |
|  |  |  |  |  |  |  | … |  |  |  |  |
|  | Category 7 - Clin + (Clinical information that its presence helped you make the diagnosis of TMA) |  | **KB-10** | **KB-11** | **KB-13** | **KB-15** | … | **ST-1** | **ST-2** | **ST-3** | **ST-4** |
|  | 7A - accelerated/malignant hypertension/Recurrent attacks of malignant Hypertension |  | 0,00% | 4,35% | 0,00% | 0,00% | … | 0,00% | 0,00% | 0,00% | 0,00% |
|  | 7B - evidence of (or triggered by) STEC (Shiga toxin-producing E Coli) infection |  | 0,00% | 0,00% | 0,00% | 0,00% | … | 0,00% | 0,00% | 0,00% | 0,00% |
|  | 7C - evidence of thrombi in any other organs/ systemic thrombosis |  | 0,00% | 8,70% | 0,00% | 4,35% | … | 8,70% | 0,00% | 8,70% | 0,00% |
|  | 7D - past history of TMA or HUS/aHUS or TTP |  | 0,00% | 0,00% | 0,00% | 0,00% | … | 0,00% | 0,00% | 65,22% | 0,00% |
|  | 7E - pregnancy/ post-partum or history of Pre-eclampsia/ Eclampsia/ HELLP syndrome |  | 0,00% | 0,00% | 0,00% | 0,00% | … | 0,00% | 0,00% | 4,35% | 0,00% |
|  | 7F - history of Anti-phospholipid syndrome |  | 0,00% | 0,00% | 8,70% | 0,00% | … | 0,00% | 0,00% | 0,00% | 4,35% |
|  | 7G - history of using drugs that are associated with TMA, such as Opana, Mitomycin M, ticlopidine, Ribavirin, Interferon |  | 0,00% | 4,35% | 0,00% | 0,00% | … | 0,00% | 0,00% | 0,00% | 0,00% |
|  | 7H - bloody diarrhea |  | 0,00% | 4,35% | 0,00% | 0,00% | … | 0,00% | 0,00% | 0,00% | 0,00% |
|  | 7I - family history of TMA |  | 0,00% | 4,35% | 0,00% | 0,00% | … | 0,00% | 0,00% | 0,00% | 0,00% |
|  | 7J - history of Scleroderma |  | 95,65% | 91,30% | 82,61% | 91,30% | … | 91,30% | 100,00% | 21,74% | 95,65% |
|  | N/A |  | 0,00% | 0,00% | 0,00% | 0,00% | … | 0,00% | 0,00% | 0,00% | 0,00% |
|  |  |  |  |  |  |  | … |  |  |  |  |
|  | Category 8 - Clin - (Clinical information that its presence helped you rule out the diagnosis of TMA) |  | **KB-10** | **KB-11** | **KB-13** | **KB-15** | … | **ST-1** | **ST-2** | **ST-3** | **ST-4** |
|  | 8A - *there are no clinical info that can help you rule out TMA |  | 47,83% | 43,48% | 47,83% | 56,52% | … | 47,83% | 52,17% | 56,52% | 60,87% |
|  | N/A |  | 0,00% | 0,00% | 0,00% | 0,00% | … | 0,00% | 0,00% | 0,00% | 0,00% |
|  |  |  |  |  |  |  | … |  |  |  |  |
|  | Category 9 - Lab + (Laboratory information that its presence helped you make the diagnosis of TMA) |  | **KB-10** | **KB-11** | **KB-13** | **KB-15** | … | **ST-1** | **ST-2** | **ST-3** | **ST-4** |
|  | 9A - schistocytes on peripheral smear (hemolytic anemia) |  | 0,00% | 4,35% | 4,35% | 4,35% | … | 0,00% | 0,00% | 13,04% | 4,35% |
|  | 9B - low haptoglobin levels/(added by 14: in the absence of history of recent transfusion) |  | 0,00% | 4,35% | 4,35% | 0,00% | … | 0,00% | 0,00% | 0,00% | 0,00% |
|  | 9C - low ADAMTS13 activity/(less than 5-10% ) |  | 0,00% | 4,35% | 0,00% | 0,00% | … | 0,00% | 0,00% | 0,00% | 0,00% |
|  | 9D - serology or cultures supporting Shiga toxin-producing E. Coli (STEC) |  | 0,00% | 0,00% | 0,00% | 0,00% | … | 0,00% | 0,00% | 0,00% | 0,00% |
|  | 9E - antibody to CFH |  | 0,00% | 0,00% | 0,00% | 0,00% | … | 0,00% | 0,00% | 0,00% | 0,00% |
|  | 9F - antibody to CFI |  | 0,00% | 0,00% | 0,00% | 0,00% | … | 0,00% | 0,00% | 0,00% | 0,00% |
|  | 9G - presence of anti-cardiolipin antibodies / lupus anti-coagulant |  | 0,00% | 4,35% | 0,00% | 4,35% | … | 0,00% | 0,00% | 47,83% | 8,70% |
|  | 9H - anemia/ hemolytic anemia/ dropping hematocrit |  | 0,00% | 4,35% | 0,00% | 0,00% | … | 0,00% | 0,00% | 0,00% | 0,00% |
|  | 9I - antibodies: APLS, ADAMTS 13, SLE |  | 100,00% | 95,65% | 95,65% | 91,30% | … | 100,00% | 100,00% | 52,17% | 91,30% |
|  | N/A |  | 0,00% | 0,00% | 0,00% | 0,00% | … | 0,00% | 0,00% | 0,00% | 0,00% |
|  |  |  |  |  |  |  | … |  |  |  |  |
|  | Category 11 - Gen + (Genetic information that its presence helped you rule out the diagnosis of TMA) |  | **KB-10** | **KB-11** | **KB-13** | **KB-15** | … | **ST-1** | **ST-2** | **ST-3** | **ST-4** |
|  | 11A - mutation in ADAMST13 gene |  | 0,00% | 0,00% | 0,00% | 4,35% | … | 0,00% | 0,00% | 0,00% | 0,00% |
|  | 11B - CFH (R1-5) (MUTATION analysis, risk haplotype determination and copy-number variation determination) |  | 0,00% | 0,00% | 0,00% | 0,00% | … | 0,00% | 0,00% | 0,00% | 0,00% |
|  | 11C - C3 gene mutation |  | 0,00% | 0,00% | 0,00% | 0,00% | … | 4,35% | 0,00% | 0,00% | 0,00% |
|  | 11D - CFI (MUTATION analysis, risk haplotype determination and copy-number variation determination) |  | 100,00% | 95,65% | 95,65% | 95,65% | … | 95,65% | 100,00% | 100,00% | 100,00% |
|  | N/A |  | 0,00% | 0,00% | 0,00% | 0,00% | … | 0,00% | 0,00% | 0,00% | 0,00% |
|  |  |  |  |  |  |  | … |  |  |  |  |
|  | Category 12 - DD# (Differential diagnosis that you considered for this case) |  | **KB-10** | **KB-11** | **KB-13** | **KB-15** | … | **ST-1** | **ST-2** | **ST-3** | **ST-4** |
|  | 12A - Donor-related TMA: TMA in the donor/ implantation biopsy |  | 13,04% | 8,70% | 4,35% | 30,43% | … | 4,35% | 0,00% | 0,00% | 0,00% |
|  | 12B - Disseminated intravascular coagulation (on preimplantatory biopsies) |  | 95,65% | 52,17% | 21,74% | 34,78% | … | 47,83% | 21,74% | 26,09% | 86,96% |
|  | 12C - Antibody-mediated rejection (acute and chronic) |  | 8,70% | 56,52% | 34,78% | 47,83% | … | 30,43% | 13,04% | 73,91% | 30,43% |
|  | 12D - TTP/ Aquired HUS /familial HUS/aHUS |  | 13,04% | 13,04% | 0,00% | 52,17% | … | 0,00% | 0,00% | 0,00% | 4,35% |
|  | 12E - Donor-related TMA: TMA seen in the allograft in the first week post Tx |  | 30,43% | 4,35% | 39,13% | 17,39% | … | 0,00% | 0,00% | 4,35% | 0,00% |
|  | 12F - Donor-related TMA: TMA seen in the allograft in the first month post Tx |  | 0,00% | 4,35% | 0,00% | 17,39% | … | 0,00% | 0,00% | 0,00% | 0,00% |
|  | 12G - Renal vein thrombosis |  | 4,35% | 0,00% | 0,00% | 4,35% | … | 0,00% | 0,00% | 4,35% | 8,70% |
|  | 12H - Hyperacute rejection |  | 0,00% | 34,78% | 8,70% | 34,78% | … | 0,00% | 0,00% | 21,74% | 17,39% |
|  | 12I - Anti-phospolipid syndrome |  | 0,00% | 0,00% | 0,00% | 0,00% | … | 69,57% | 17,39% | 0,00% | 0,00% |
|  | 12J - Chronic TX Glomerulopathy |  | 4,35% | 26,09% | 13,04% | 8,70% | … | 13,04% | 69,57% | 17,39% | 13,04% |
|  | N/A |  | 0,00% | 0,00% | 0,00% | 0,00% | … | 0,00% | 0,00% | 0,00% | 0,00% |
