# Supplementary material for: Delphi: A Democratic and Cost-Effective Method of Consensus Generation in Transplantation
Source: Transpl Int. 2023 Aug 23;36:11589. doi: 10.3389/ti.2023.11589 (PMC10481336; doi:10.3389/ti.2023.11589)
Supplement: Supplementary file 3 [file Table2.DOCX]

**Supplemental table S2.**

| Category | Criteria sub-group | Criteria # |
| --- | --- | --- |
| 1 | LM + | 11 |
| 2 | LM - | 0 |
| 3 | IF + | 1 |
| 4 | IF - | 2 |
| 5 | EM + | 4 |
| 6 | EM - | 0 |
| 7 | Clin + | 2 |
| 8 | Clin - | 0 |
| 9 | Lab + | 4 |
| 10 | Lab - | 4 |
| 11 | Gen | 0 |
| 12 | Differential diagnosis | 8 |
| Definitions | 4 LM+ and 4 EM+ | 8 |
